# An Unusual Case of Severe Vitamin B12 Deficiency With Early Erythroblasts on Peripheral Blood Film

**DOI:** 10.1002/jha2.70178

**Published:** 2025-11-04

**Authors:** Jessica Muscat, Alexander Gatt

**Affiliations:** ^1^ Haematology Department Mater Dei Hospital Msida Malta

1

A 72‐year‐old lady presented to the emergency department with extreme pallor and exertional dyspnoea. Her medical history was unremarkable, and she was not on any regular medications.

Initial full blood count (FBC) revealed a haemoglobin of 50 g/L (reference range: 120–155 g/L), a mean cell volume of 126.5 fL (reference range: 79.0–90.6 fL), a borderline reticulocyte count of 20 × 10^9^/L (reference range: 20–79 × 10^9^/L) and thrombocytopenia of 49 × 10^9^/L (reference range: 132–345 × 10^9^/L). Total white cell count and differential were within normal limits. The patient was admitted for investigation of these cytopenias.

The peripheral blood film received in the laboratory was regrettably obtained post‐transfusion, demonstrating a dimorphic picture (Figure 1). Features of megaloblastic anaemia were observed, such as macro‐ovalocytes and hypersegmented neutrophils (Panel A), macropolycytes (Panel B) and occasional apoptotic neutrophils (Panel C) [1]. In addition, there was an unexpected number of circulating nucleated red cell precursors with left shift, an atypical finding on peripheral blood in the context of megaloblastic anaemia [2]. These precursors consisted of occasional basophilic erythroblasts, characterized by basophilic cytoplasm and a perinuclear halo (Panels A and D); polychromatic erythroblasts, with less intense basophilic cytoplasm and a more condensed chromatin pattern (Panels E and F); and numerous abnormal orthochromatic erythroblasts (Panel G).

**FIGURE 1:**
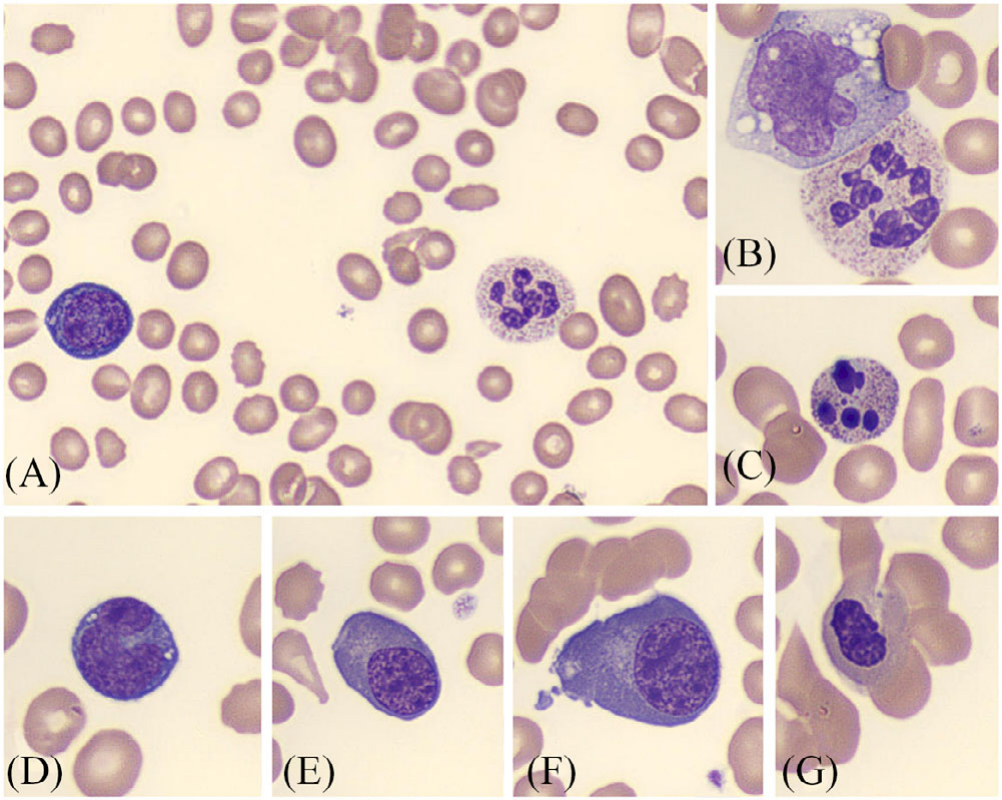
(A–G) Peripheral blood smear (May–Grünwald Geimsa; 100 ×objective). Panel (A) Dimporphic picture showing features of megaloblastic anaemia such as macro‐ovalocytes, a hypersegmented neutrophil, and an unexpected circulating basophilic erythroblast. Panel (B) Macropolycyte next to a monocyte. Panel (C) Apoptotic neutrophil. Panel (D) Another basophilic erythroblast, characterized by dark blue cytoplasm and a perinuclear halo. Panels (E) and (F) Polychromatic erythroblasts that have lighter, more abundant cytoplasm, and nuclei with a more condensed chromatin pattern. Panel (G) Orthochromatic erythroblast with an irregularly shaped nucleus.

Haematinic assessment confirmed severe vitamin B12 deficiency (< 111 pmol/L; reference range: 142–725 pmol/L), with normal folate levels (8.75 nmol/L; reference range: 6–39 nmol/L). Iron studies, ferritin and serum protein electrophoresis were unremarkable. Haemolytic anaemia was excluded based on a low reticulocyte count, normal bilirubin and LDH, and absence of schistocytes, red cell agglutinates or spherocytes on blood film.

The patient did not have neurological features of vitamin B12 deficiency, such as ataxia, paresthesia and neuropsychiatric changes. She followed an omnivorous diet and did not have a history of gastrointestinal surgery or malabsorption. Tissue transglutaminase levels were normal, and a CT scan revealed no abnormalities that could account for the vitamin B12 deficiency.

Treatment with intramuscular hydroxocobalamin injections was initiated, along with regular monitoring of the complete blood count and potassium levels. Due to the unusual presence of early red cell precursors, repeat peripheral blood films were advised during hydroxocobalamin therapy. Persistence of these precursors would have prompted consideration of a bone marrow biopsy to exclude diagnoses such as acute leukemia.

The patient achieved near normalization of her counts within 2 weeks, with a haemoglobin of 105 g/L, and a platelet count of 123 × 10^9^/L. A repeat blood film at this point showed significant improvement, including a reduction in macrocytes, normal neutrophil nuclear segmentation and absence of the circulating early red cell precursors.

Subsequent investigations revealed an intrinsic factor antibody level of 173.5 U/mL (reference range: 0.0–20.0 U/mL) with moderate positivity for anti‐gastric parietal cell antibody, confirming a diagnosis of pernicious anaemia. Serial follow‐up blood counts showed sustained normal blood count indices, with no alarming features on blood films. In view of the good response to therapy and confirmatory serology for pernicious anaemia, bone marrow examination was deemed unnecessary, and the patient was maintained on lifelong cobalamin therapy.

## Author Contributions

J.M. captured the images and wrote the manuscript. A.G. reviewed and edited the manuscript. Both authors approved the final version of the manuscript.

## Funding

The authors have nothing to report.

## Ethics Statement

The authors have nothing to report.

## Consent

The authors have nothing to report.

## Conflicts of Interest

The authors declare no conflicts of interest.

## Data Availability

All data relevant to this publication are included within the article.
